# Recombinant Haemagglutinin Derived From the Ciliated Protozoan *Tetrahymena thermophila* Is Protective Against Influenza Infection

**DOI:** 10.3389/fimmu.2019.02661

**Published:** 2019-11-13

**Authors:** Karina Jawinski, Marcus Hartmann, Charanjit Singh, Ekaterina Kinnear, David C. Busse, Annalisa Ciabattini, Fabio Fiorino, Donata Medaglini, Claudia Maria Trombetta, Emanuele Montomoli, Vanessa Contreras, Roger Le Grand, Celine Coiffier, Charlotte Primard, Bernard Verrier, John S. Tregoning

**Affiliations:** ^1^Cilian AG, Munster, Germany; ^2^Department of Infectious Disease, St Mary's Campus, Imperial College London, London, United Kingdom; ^3^Laboratory of Molecular Microbiology and Biotechnology, Department of Medical Biotechnologies, University of Siena, Siena, Italy; ^4^Department of Molecular and Developmental Medicine, University of Siena, Siena, Italy; ^5^VisMederi s.r.l., Siena, Italy; ^6^CEA-Université Paris Sud 11-INSERM U1184, Immunology of Viral Infections and Autoimmune Diseases, IDMIT Department, IBFJ, Le Kremlin-Bicêtre, France; ^7^Laboratoire de Biologie Tissulaire et d'Ingénierie Thérapeutique, UMR 5305, Université Lyon 1, CNRS, IBCP, Lyon, France; ^8^Adjuvatis, Lyon, France

**Keywords:** influena virus, protozoa, adjuvant, nanoparticle, vaccine manufacture

## Abstract

Current influenza vaccines manufactured using eggs have considerable limitations, both in terms of scale up production and the potential impact passaging through eggs can have on the antigenicity of the vaccine virus strains. Alternative methods of manufacture are required, particularly in the context of an emerging pandemic strain. Here we explore the production of recombinant influenza haemagglutinin using the ciliated protozoan *Tetrahymena thermophila*. For the first time we were able to produce haemagglutinin from both seasonal influenza A and B strains. This ciliate derived material was immunogenic, inducing an antibody response in both mice and non-human primates. Mice immunized with ciliate derived haemagglutinin were protected against challenge with homologous influenza A or B viruses. The antigen could also be combined with submicron particles containing a Nod2 ligand, significantly boosting the immune response and reducing the dose of antigen required. Thus, we show that *Tetrahymena* can be used as a manufacturing platform for viral vaccine antigens.

## Introduction

Influenza infections are one of the most common causes of primary care consultation and represent an important economic burden worldwide (1). The World Health Organization (WHO) estimates influenza epidemics to result in about 3 to 5 million cases of severe illness globally each year, leading to 290,000 to 650,000 deaths. Annual influenza vaccination is considered by the WHO to be the most effective strategy to prevent disease caused by the influenza A and B viruses currently co-circulating in humans. Vaccination is recommended for people aged over 18 years in the USA, and over 65 in most of European States (2).

To date, most commercially available flu vaccines are produced using embryonated chicken eggs (3). However, egg-based influenza vaccine production is complex to scale up and work intensive, taking several months following the isolation of new strains. Growth of viruses in eggs may lead to a selection pressure, altering antigenicity (4), particularly for the heavily humanized H3N2 strains (5). Mutations induced by growth in eggs might change viral antigenicity and in consequence could contribute to a lower than anticipated vaccine efficacy (6). Furthermore, eggs may not be appropriate for the growth of highly pathogenic avian strains, where there may be a limited supply of embryonated eggs due to effects of influenza virus on poultry (7).

Alternative approaches that can generate safe and effective vaccines are needed in addition to egg–based vaccines (8). The focus has been on cell culture-based technologies for mass production of influenza vaccines and there is a licensed cell culture-based vaccine: Flucelvax™ from Seqirus (Holly Springs, NC). Cell-based technologies offer advantages over egg produced vaccines. A modest improvement of vaccine efficacy was seen for inactivated cell culture-grown vaccines compared to egg-based vaccines in subjects 65 years of age and older (9). Completely cell cultured virus in influenza vaccines, which was not propagated in eggs, can avoid the egg-adaptation changes of the influenza virus (10). But cell lines can induce mutations, reducing vaccine efficacy (11). Furthermore, cell culture-based technologies using mammalian systems for manufacturing of influenza vaccine from whole virus still involve chemical virus inactivation treatment, which can alter the vaccine antigenicity (12). An alternative to viral growth and inactivation is to manufacture vaccines derived from recombinant protein. For example, influenza antigens can be produced in insect cells using baculoviral vectors expressing target antigens. However, the current market share of this approved recombinant influenza antigen produced in insect cells (Flublok™ from Sanofi, Swiftwater, PA) is relatively low at 1–2%.

Other approaches for the production of recombinant influenza antigen are being investigated, for example the expression of haemagglutinin (HA) in bacterial cells (13) or in the ciliate *Tetrahymena thermophila*. The freshwater ciliated protozoan *T. thermophila* is one of the best characterized unicellular eukaryotic organisms (14). Although ciliates have been extensively used as a model system in molecular and cell biology, their application as a biopharmaceutical manufacturing platform remains underexplored (15, 16). *T. thermophila* has a number of advantages as a biotechnological expression system, cells grow rapidly to high densities in simple, inexpensive media. The fermentation process uses conventional equipment, including bioreactors and down-stream processing plant, typically used for *Escherichia coli* or yeast systems. The whole bio-process is readily up-scalable for large volumes (17). *T. thermophila* has been used for the expression of recombinant proteins, which can be used as candidates for vaccines against protozoan pathogenic agents, for example the malaria agent *Plasmodium falciparum* (18). Furthermore, it has been shown, that *T. thermophila* is also suitable as expression host for the recombinant production of influenza virus proteins (19). One other consideration is post-translational modification, as a eukaryotic organism *T. thermophila* is able to post-translational modify proteins by glycosylation or formation of disulfid bridges (20). The naturally occurring generalized N-glycan structure of secreted proteins by *Tetrahymena* is described as a biantennary non-complex oligomannose-type Man3GlcNAc2 structure with limited heterogeneity (16).

Here we describe the production of a recombinant influenza subunit vaccine by overexpression of the surface protein HA from influenza virus A and B strains using the ciliate *T. thermophila* as expression system. Purified recombinant HA (rHA) was evaluated *in vivo* in a non-human primate case study as well as in a mouse model. Since the use of adjuvants or delivery systems can be a strategy for increasing antigen immunogenicity and minimize the dose of vaccine necessary to confer immunity (3, 21), we also combined the influenza antigens with PLA-Nod2 particles, a promising vaccine vehicle (22, 23). We show that the recombinant vaccine produced using the ciliate expression system was immunogenic in non-human primates and mice as well as protective in a mice challenge model. Furthermore, we demonstrate dose sparing when HA antigens were combined with PLA-Nod2 particles.

## Materials and Methods

### Constructs

Synthetic genes for the full-length HA (containing the sequences of HA1 and HA2 including the transmembrane region and the cytoplasmic tale) of influenza virus A/California/07/2009 (A/Cal; accession # EPI177294, 567 amino acids), A/New Caledonia/20/99 (A/NC; accession # EPI139303, 565 amino acids), A/Uruguay/716/2009 (A/Uru; accession # EPI152544, 567 amino acids), B/Brisbane/60/2008 (B/Bri; accession # EPI394898, 586 amino acids), B/Jiangsu/10/2003 (B/Jia; accession # EPI242836, 584 amino acids) and B/Malaysia/2506/04 (B/Mal; accession # EPI175755, 585 amino acids) (https://www.gisaid.org/) were codon-optimized (GeneArt^®^, Life Technologies™ Cooperation). The synthetic genes were each cloned into a modified version of the integrative expression vector pKOIX where integration flanks were replaced by sequences of the GRL3 locus of *T. thermophila* according to methods described in Weide et al. (Details on vector are available at Cilian AG) (20). Final expression cassette carried a 1 kb fragment of the cadmium-inducible *MTT1* promoter-active region (24), BTU2-terminator, and the corresponding HA gene.

### Strains, Transformation, and Cultivation of *T. thermophila*

*T. thermophila* inbred strains (B1868/4, B1868/7, B2086/1, and SB1969; available at Tetrahymena Stock Center or American Type Culture Collection) were used as transformation hosts. Conjugating cells were transformed with the integrative expression vectors *via* biolistic bombardment using standard protocols (25, 26). Individual transformants were routinely cultivated at 30°C without agitation in 1.5 ml SPO medium (1% potato peptone, 0.5% yeast extract, 0.1% ferrous sulfate chelate solution, 0.2% glucose) or in SPP medium (1% proteose peptone, 0.5% yeast extract, 0.1% ferrous sulfate chelate solution, 0.2% glucose). The antibiotic Paromomycin (500 μg/ml) was added to each media for several passages to support the allelic assortment process. Assorted transformants were cultivated in 1.5 ml scale without antibiotic at 30°C and 80 rpm in a Multitron AJ incubation shaker (Infors).

**Figure 1:**
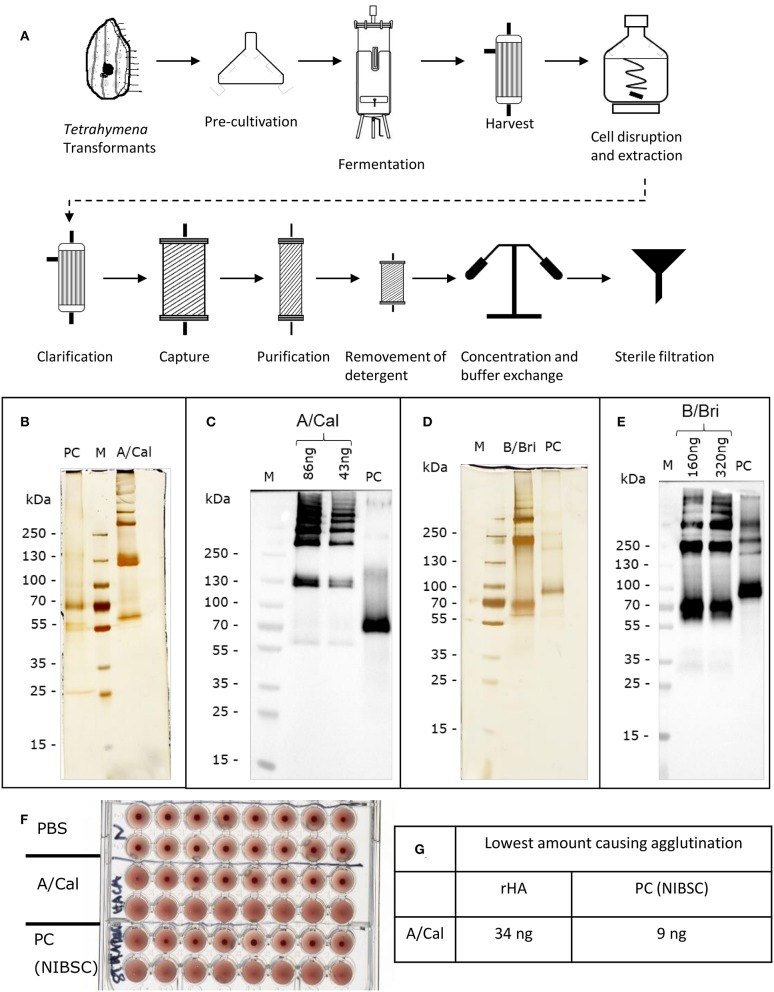
Production and characterization of recombinant rHA expressed in *Tetrahymena thermophila*. **(A)** Schematic of production and purification; see Table S1 for details of conditions used for different rHA. Each purified rHA was analyzed by SDS-PAGE and silver staining **(B,D)** or Western blot **(C,E)**. A/Cal, ciliate derived rHA A/California/07/09; B/Bri, ciliate derived rHA B/Brisbane/60/2008; PC, positive control either A/California/07/09 antigen (12/168, NIBSC) or B/Brisbane/60/2008 (13/234, NIBSC) as appropriate; M, molecular weight marker. **(F)** Example haemagglutination assay of rHA A/Cal, samples two-fold diluted, **(G)** Example haemagglutination value for rHA A/Cal.

HA from the A/California, A/New Caledonia, A/Uruguay, B/Brisbane B/Jiangsu and B/Malaysia virus strains were all produced in *T. thermophila*. The production and purification process are summarized in Figure 1A.

Transformants bearing the A/Cal, B/Bri, B/Jia, or B/Mal antigen expression module were cultivated in 400 ml SPO or SPP medium supplemented with 1% Casein hydrolysate at 30°C and 80 rpm in Erlenmeyer flasks (Fernbach design; 1,800 ml). Expression of each antigen was induced by addition of 94 μM Cadmium (CdCl_2_, Sigma-Aldrich) to a logarithmically growing culture. Cell harvest was performed 15–17 h post-induction by centrifugation at 15°C for 8 min at 1,200 × g. Cell pellet was resuspended in 20 mM sodium phosphate buffer supplemented with 2.5 mg/lE-64 (PeptaNova) and stored at −80°C.

For pilot scale fermentation transformants bearing the A/NC or A/Uru antigen expression module were cultivated in a UD 50 L-fermenter (Biostat^®^; Sartorius) in 50 L of SPP medium supplemented with 1% Casein hydrolysate. The temperature was maintained at 30°C and pO_2_ was controlled at 20% of the air saturation level. The pH value was regulated to pH 7.0. Expression of each antigen was induced by addition of 47 μM Cadmium (CdCl_2_, Sigma-Aldrich) to a logarithmically growing culture. Cell harvest was performed 15–17 h post-induction by using a hollow fiber module (Spectrum; 0.5 μm; polyethersulfone). Cells were washed once with 20 mM sodium phosphate buffer supplemented with 2.5 mg/L E-64 and stored at −80°C.

### Purification Procedure

The fermentation and purification process slightly varied per HA of different viral strains. Detail of the A/Cal process is given here, variations and the buffers details are described in Table S1.

The extraction of the rHA from disrupted cells took place using buffer A by stirring on a magnetic stirrer at 4°C overnight. The extract was clarified by using a hollow fiber module (GE Healthcare; 0.45 μm; polyethersulfone). The pH of the filtrate was adjusted to pH 7.13 by using buffer B. The filtrate was loaded on Capto SP ImpRes (equilibrated with buffer C) at 6.5 ml/min. After loading, the column was washed with buffer C. rHA was eluted with buffer D. The eluate was concentrated and diafiltrated with buffer E by cross flow filtration (Sartorius; MWCO 50 kDa; polyethersulfone). The solution was loaded on a Fetuin-Agarose column (equilibrated with pre-cooled buffer F) at 1.5 ml/min. The column was washed with pre-cooled buffer F. The rHA was eluted from the Fetuin-Agarose column with buffer G at room temperature and rHA preparation was concentrated and diafiltrated with buffer H by cross flow filtration (MWCO 50 kDa). The solution was loaded on Capto SP ImpRes HiScreen (equilibrated with buffer I) at 1.6 ml/min. After loading, the column was washed with buffer I. rHA was eluted with buffer J. The eluate was concentrated and diafiltrated with buffer K at 16°C by a centrifugal concentrator (Sartorius; MWCO 50 kDa; polyethersulfone) to a final concentration of 0.86 mg/ml (Pierce™ BCA Protein Assay Kit, Thermo Scientific). In a final step the solution was filtered using a sterile filter (Carl Roth; 0.22 μm, polyvinylidene fluoride).

### SDS-Page, Western Blot Analysis, and Silver Staining

Protein expression was verified by sodium dodecyl sulfate polyacrylamide gel electrophoresis (SDS-PAGE) on 4–20% bis-tris gels (Anamed Elektrophorese) (27). The samples were treated with a sample buffer containing 4% SDS, no reducing agent was added. The gels were either blotted onto nitrocellulose membranes or stained according to Pierce^®^ Silver Stain Kit (Thermo Scientific). Blotted nitrocellulose membranes were blocked in phosphate buffered saline (PBS) containing 0.05% Tween 20 and 1% skim milk (PBS-TM) for A/Cal, B/Bri. Expression of respective recombinant HA in transformed ciliates was detected by an appropriate polyclonal sheep anti-Influenza HA from NIBSC (anti-A/California/07/2009-HA serum−14/310, NIBSC: anti-B/Brisbane/60/2008-HA serum−13/254, NIBSC) and a horse radish peroxidase (HRP)-conjugated rabbit-anti-sheep IgG secondary antibody. The blots were developed using chemiluminescence and visualized using a Fusion imaging system and software (Peqlab).

### Haemagglutination Assay to Assess Antigen Quality

Haemagglutination assay was performed using guinea pig erythrocytes for A/Cal (1% suspension) and chicken erythrocytes for A/NC, A/Uru, B/Bri, B/Jia, and B/Mal (0.5% or 1% suspension). Erythrocytes were washed and adjusted to the corresponding concentration using PBS supplemented with 0.05% BSA (pH 7.4). Purified rHA samples were titrated in a 96-well microtiter plate with V-shaped wells (Carl Roth) and incubated at 4°C for 1 h.

### Non-human Primates Study Immunizations and Sampling

Groups of 2 macaques were immunized on weeks 0, 3, and 6 by either intramuscular (i.m.) route (group 1) or subcutaneous (s.c.) (group 2) injection with 45 μg of each rHA A/NC, A/Uru, B/Jia, and B/Mal. Blood samples were collected on week 0, 3, 4, 6, 7, and 10 to analyse the specific serum-antibody response.

### Poly Lactic Acid—Nod2 Particles Synthesis and Characterization

i-Particles^®^ from Adjuvatis (France) are PLA particles made by nanoprecipitation method by adding dropwise a solution of poly-D,L-lactic acid polymer dissolved on acetone (2% w/v) to an aqueous phase composed of water and ethanol, under moderate stirring (250 rpm). No surfactant or stabilizer was required to stabilize the colloid solution. Solvents were removed by evaporation under reduced pressure using a Buchi rotavapor including a water bath at 30–34°C and a cooler maintained at −10°C. The final particles concentration was around 7% level of solid and was precisely determined by weighing the wet and dried materials. PLA NP-Nod2 also provided by Adjuvatis were made by nanoprecipitation technique as described previously (23). The Nod2 ligand, Mifamurtide, is synthetic derivative of muramyl dipeptide (Sigma-Aldrich). This ligand was added to the PLA-acetone solution at a mass ratio of 1% w/w ligand: PLA.

Nod2 ligand encapsulation efficiency was obtained through determination of the amount of remaining free ligand in the supernatant after centrifugations of the nanoparticle solution (10 min at 10,000 × g). Supernatants were cultured with HEK-Blue™-hNOD reporter cell lines (InvivoGen) in 96-microwell plates (50,000 cells/well in duplicate) to study the stimulation of Nod2 receptor by monitoring activation of NF-κB pathway which induces the production of secreted embryonic alkaline phosphatase (SEAP) and the use of a detection medium that turns blue in the presence of alkaline phosphatase. The absorbance of the samples was measured at 650 nm using a microplate reader (ThermoScientific). Nod2 ligand solutions were initially assayed with HEK-Blue™-hNOD Cells to plot the calibration curve. The encapsulation efficiency of Nod2 ligands was calculated by the ratio of the ligand mass in NPs over the total mass of ligand in the recipe.

### HA Antigen Adsorption Onto PLA-Nod2 Particles

HA from *Tetrahymena* was adsorbed on PLA-Nod2 particles by mixing equal volumes of particles dispersion (diluted in water at 3% level of solid) and protein solution (at 30 μg/ml) with moderate end-overhead stirring, for 2 h at room temperature. At the end of incubation, a fraction of HA-coated particles was removed for characterization. Four hundred microliters were high-speed centrifuged (10 min at 10,000 × g) and supernatant containing the non-adsorbed HA fraction was quantified (Pierce™ BCA Protein Assay Kit) to deduce the adsorbed HA concentration. Concentrated sterile NaCl solution was added to HA-particles solutions to adjust the osmole concentration to 300 milli-osmole and the antigen concentration to 90 μg/ml of vaccine formulations. Hydrodynamic diameter, size distribution and Zeta potential of formulated particles were determined. The absorbance of the samples was measured at 562 nm using a microplate reader (Multiskan FC, Thermo Scientific).

### Mouse Immunization

For experiments aimed to characterize the antibody response in mice, 7 weeks old female BALB/c mice were purchased from Charles River (Lecco, Italy) and maintained in cages provided with food and water *ad libitum*, with environmental enrichment and nesting. Groups of 6 mice were immunized i.m. and s.c. with 15 μg/mouse of rHA A/Cal in the volume of 50 μl/mouse, or with 30 μg/mouse of rHA B/Bri in the volume of 100 μl/mouse, respectively. The vaccines were administered three times at 3-week interval (week 0, 3, 6). Blood samples were taken from the temporal plexus of individual mice on weeks −1, 3, 6, and 8, incubated for 30 min at 37°C and centrifuged at 1,200 × g at 4°C for 15 min for collecting sera that were stored at −80°C.

For experiments to test protection against infection, 6–10 week old female CB6F1 mice were obtained from Harlan UK Ltd (Barking, UK) and kept in specific-pathogen-free. Studies followed the ARRIVE guidelines. Mice were immunized i.m. with 1.5 or 15 μg ciliate derived rHA A/Cal or with 3 or 30 μg rHA B/Bri alone in 50 μl in a prime-boost-boost regimen. 1.5 μg egg derived A/Cal HA (GSK, Siena, Italy) was used as a positive control. Where used, NOD particles were combined with 0.015 μg rHA A/Cal and delivered s.c. For infections, mice were anesthetized using isoflurane and infected i.n. with 100 μl influenza virus or sterile PBS. Mice were culled using 100 μl intraperitoneal pentobarbitone (20 mg dose, Pentoject, Animalcare Ltd. UK) and tissues collected as previously described (28). Viruses were propagated in Madin-Darby Canine Kidney (MDCK) cells, in serum-free DMEM supplemented with 1 μg/ml trypsin. Influenza viral load was assessed by PCR as described previously (29). Clinical score was assessed as described previously (30). Challenge dose of virus was titrated (30) to minimize animal suffering, specifically it was a non-lethal model, to match refinement guidelines.

### Influenza Viruses

The influenza infectious viruses used for HA inhibition, virus neutralization *in vitro* assays and *in vivo* challenge were seasonal influenza strains obtained from NIBSC: A/California/7/2009 (H1N1, 15/252) and B/Brisbane/60/2008 (Victoria lineage, 15/146). The viruses were propagated in 11 days embryonated chicken eggs.

### Haemagglutination Inhibition Assay

All serum samples, including sheep hyper-immune sera (A/California/7/2009: NIBSC code 09/152, B/Brisbane/60/2008: NIBSC code 11/136) as positive controls and human serum without IgA, IgM, and IgG as negative control (Sigma-Aldrich, S5393), were pre-treated with receptor destroying enzyme (RDE) (ratio 1:5) from Vibrio Cholerae (Sigma Aldrich) for 18 h at 37°C in a water bath and then heat inactivated for 1 h at 56°C in a water bath with 8% sodium citrate (ratio 1:4). Turkey red blood cells were centrifuged two times, washed with a saline solution (0.9%) and adjusted to a final dilution of 0.35%. Serum samples were two-fold diluted in duplicate with saline solution (0.9%) in a 96-wells plate from an initial dilution of 1:10. Twenty-five microliters of standardized viral antigen (4 HA units/25 μl) were added to each well and the mixture was incubated at room temperature for 1 h. Turkey red blood cells were added and after 1 h incubation at room temperature, the plates were evaluated for presence of agglutination inhibition. The antibody titer is expressed as the reciprocal of the highest serum dilution showing complete inhibition of agglutination. Since the starting dilution is 1:10, the lower limit of detectable antibody titer is 10. When the titer is under the detectable threshold, the results were conventionally expressed as 5 for calculation purposes, half the lowest detection threshold.

### ELISA Assay

The A/Cal-specific and B/Bri-specific serum IgG were evaluated by enzyme-linked immunosorbent assay (ELISA) as previously described (31). Briefly, MaxiSorp microtiter plates (Nunc) were coated with recombinant A/Cal- and B/Bri- (2 μg/ml; Cilian as described above) overnight at 4°C, blocked with PBS and 1% BSA, and then added with serum samples titrated in two-fold dilutions. Samples were then incubated with the alkaline phosphatase-conjugate goat anti-mouse IgG (diluted 1:1000, Southern Biotechnology) for 1 h at 37°C and developed by adding 1 mg/ml of alkaline phosphatase substrate (Sigma-Aldrich). The optical density was recorded using Multiskan FC Microplate Photometer (Thermo Scientific).

### Virus Neutralization Assay

The MDCK cell cultures were grown at 37°C in 5% CO_2_. Serum samples, including positive and negative controls, previously heat-inactivated at 56°C for 30 min, were two-fold diluted with ultra MDCK culture medium with 2 μg/ml of trypsin from bovine pancreas (TPCK, Sigma-Aldrich) in a 96-wells plate and mixed with an equal volume of virus (100 TCID50/well). After 1 h incubation at 37°C in 5% CO_2_, the mixture was added to the MDCK cell suspension (1.5 × 10^5^ cells/ml). Plates were read for cytopathic effect after 3 days incubation at 37°C in 5% CO_2_.

### Statistical Analysis

Calculations as described in figure legends were performed using Prism 6 (GraphPad Software Inc.).

### Data Availability

All data generated or analyzed during this study are included in this published article (and it Supplementary Information Files).

### Ethics Statement

Adult cynomolgus macaques (*Macaca fascicularis*) were imported from Mauritius and housed in the facilities of the Infectious Disease Models and Innovative Therapies (IDMIT) Center (CEA, Fontenay-aux-Roses, France). Two male animals were included in each experimental group. NHPs were used at the CEA in accordance with French regulations and under the supervision of national veterinary inspectors (CEA Permit Number A 92-032-02). The CEA complies with the Standards for Human Care and Use of Laboratory Animals, as set out by the Office for Laboratory Animal Welfare (OLAW, USA) and is accredited under OLAW Assurance number #A5826-01. The use of NHP at the CEA complies with the recommendations in European Directive 2010/63 (recommendation N°9). The animals were used under the supervision of the veterinarians responsible for the animal facility. This study was approved and accredited under statement number (A15 007) by the ethics committee “Comité d'Ethique en Expérimentation Animale du CEA,” registration number 44 for the French Ministry of Research. Animals were housed in pairs in modules allowing social interaction, under controlled humidity, temperature and light conditions (12 h light/12 h dark cycles). Water was available *ad libitum*. Animals were monitored and fed 1–2 times daily with commercial monkey chow and fruit by trained personnel. Macaques were provided with environmental stimuli including toys, foodstuffs and music under the supervision of the CEA's Animal Welfare Body. Experimental procedures (animal handling, immunization protocols, and blood sampling) were conducted after sedating animals with ketamine chlorhydrate (Rhône-Mérieux, Lyon, France, 10 mg/kg).

Mouse studies in Italy were treated according to national guidelines (Decreto Legislativo 26/2014). Animals were housed under specific pathogen-free conditions in the animal facility of the Laboratory of Molecular Microbiology and Biotechnology (LA.M.M.B.), Department of Medical Biotechnologies at University of Siena, Italy. All animal studies were approved by the Italian Ministry of Health with authorization n° 1004/2015-PR, 22 Sept 2015. Mouse studies performed in the UK in accordance with the United Kingdom's Home Office guidelines and all work was approved by the Animal Welfare and Ethical Review Board (AWERB) at Imperial College London.

## Results

### Protein Expression, Purification, and Biological Characterization of Purified rHA

In order to evaluate the potential of *T. thermophila* as an expression host for production of different recombinant influenza HA expression vectors coding for full length HA, codon-optimized, synthetic genes were generated and *Tetrahymena* cells were transformed with the expression vectors. The transformed cells were cultured in up to 50 liters. rHA-bearing cells were harvested 15–17 h after induction and purified via chromatographic steps (Figure 1A). After purification, each *Tetrahymena* derived HA antigen solution was characterized for their ability to form higher order oligomers like di-, tri-, and multimers by silver staining subsequent to SDS-PAGE. The A-strain antigen A/Cal (Figure 1B) showed a single polypeptide with an apparent molecular weight of ~60 kDa and bands at ~130 kDa and >250 kDa indicating the formation of di-, tri-, and multimers. This was also observed for the A/NC and A/Uru antigens (Figure S1). For the B/Bri antigen (Figure 1D) beside a single polypeptide with an apparent molecular weight of ~70 kDa, higher order structures at ~200 and >250 kDa were visible indicating the formation of tri- and multimers of this antigen. This was also observed for the B/Jia and B/Mal antigens (Figure S1). All these bands gave a positive signal in Western blot analysis using specific antibodies (Figures 1C,E). Furthermore, *in vitro* potency of purified rHA solution was confirmed using haemagglutination assay (Figures 1F,G). Purified antigens from ciliates were able to agglutinate red blood cells indicating biological activity.

### Ciliate Produced rHA Induces an Antibody Response in Mice

**Figure 2:**
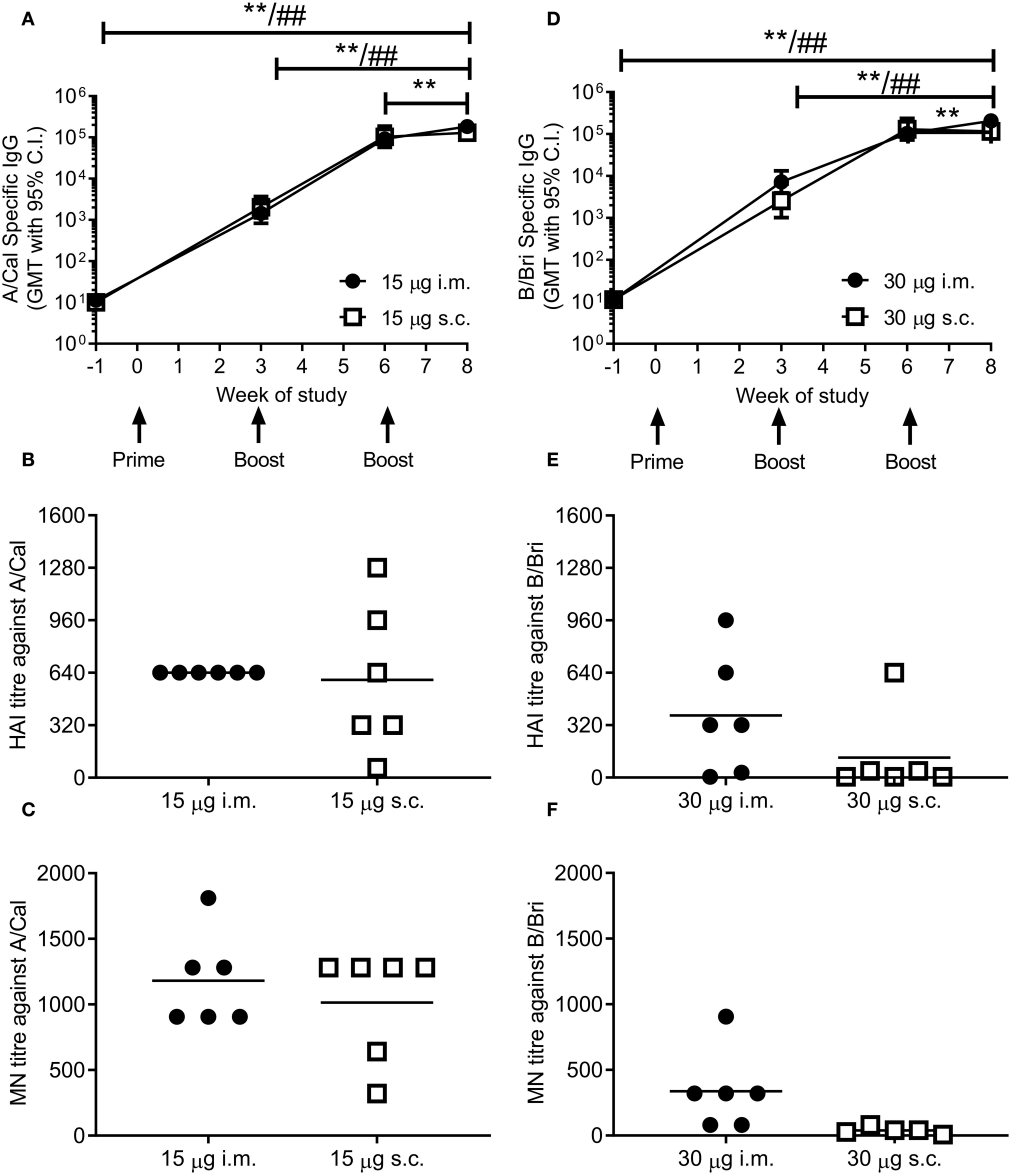
Ciliate produced rHA induces an antibody response in mice. BALB/c mice were immunized three times at 3 weekly intervals with either 15 μg A/Cal **(A–C)** or 30 μg B/Bri **(D–F)** by the intramuscular (i.m.) or subcutaneous (s.c.) routes. A/Cal-specific and B/Bri-specific IgG were evaluated in sera collected before immunization (week -1) and at weeks 3, 6 and 8, by ELISA. Values are reported as geometric mean titers (GMT) ± 95% CI of 6 mice per group **(A,D)**. Sera for haemagglutination inhibition (HAI: **B,E**) and microneutralization (MN: **C,F**) analysis were collected at week 8. Symbols represents individual mice and bars represents the mean of *n* = 6 animals **(B,C,E,F)**. ^**^p < 0.01 between i.m. at week 8 and other time points, ^*##*^*p* < 0.01 between s.c. at week 8 and other time points by ANOVA and Tukey post test for multiple comparisons.

To test whether ciliate produced rHA was immunogenic, mice were immunized in a prime-boost-boost regime with 15 μg per dose of rHA A/Cal. Mice were immunized by either the i.m. or s.c. route. A/Cal specific IgG was measured by binding ELISA at various time points after immunization (Figure 2A). Both s.c. and i.m. immunization induced significant influenza specific IgG already after the primary immunization, with GMT titers of 2,000 and 1,400, respectively (week 3, *P* < 0.05 compared to baseline). A/Cal specific IgG were increased by booster immunizations to GMTs of 103,000 and 92,000 (week 6) and 130,000 and 184,000 (week 8) after s.c. and i.m. immunizations, respectively. The quality of the antibody response was assessed by haemagglutination inhibition (HAI) assay at week 8 (Figure 2B). An HAI response was detectable after both routes of immunization, and there was no significant difference between the two routes. Likewise, H1N1 neutralizing antibodies were detectable after immunization with the ciliate derived rHA A/Cal (Figure 2C).

To confirm that the ciliate production platform could generate immunogenic material for multiple strains of influenza, the B/Bri antigen was also generated. Mice were immunized in a prime-boost-boost regime with 30 μg per dose of rHA B/Bri. Again, a significant primary response was observed upon immunization by both s.c. and i.m. routes (Figure 2D), with GMTs of 2,560 and 7,200, respectively (week 3, *P* < 0.05 compared to baseline). B/Bri specific IgG were increased by booster immunizations to GMT of 130,000 and 103,000 (week 6) and 116,000 and 206,000 (week 8) after s.c. and i.m. immunizations, respectively.

The quality of the antibody response was assessed by HAI assay at week 8 (Figure 2E). An HAI response against B/Bri was detectable after i.m. route of immunization, but not after s.c. route. Likewise, B/Bri neutralizing antibodies were detectable after i.m. immunization with the ciliate derived rHA (Figure 2F). These studies show that ciliate derived antigens are immunogenic.

### Ciliate Produced rHA Induces an Antibody Response in Non-human Primates

**Figure 3:**
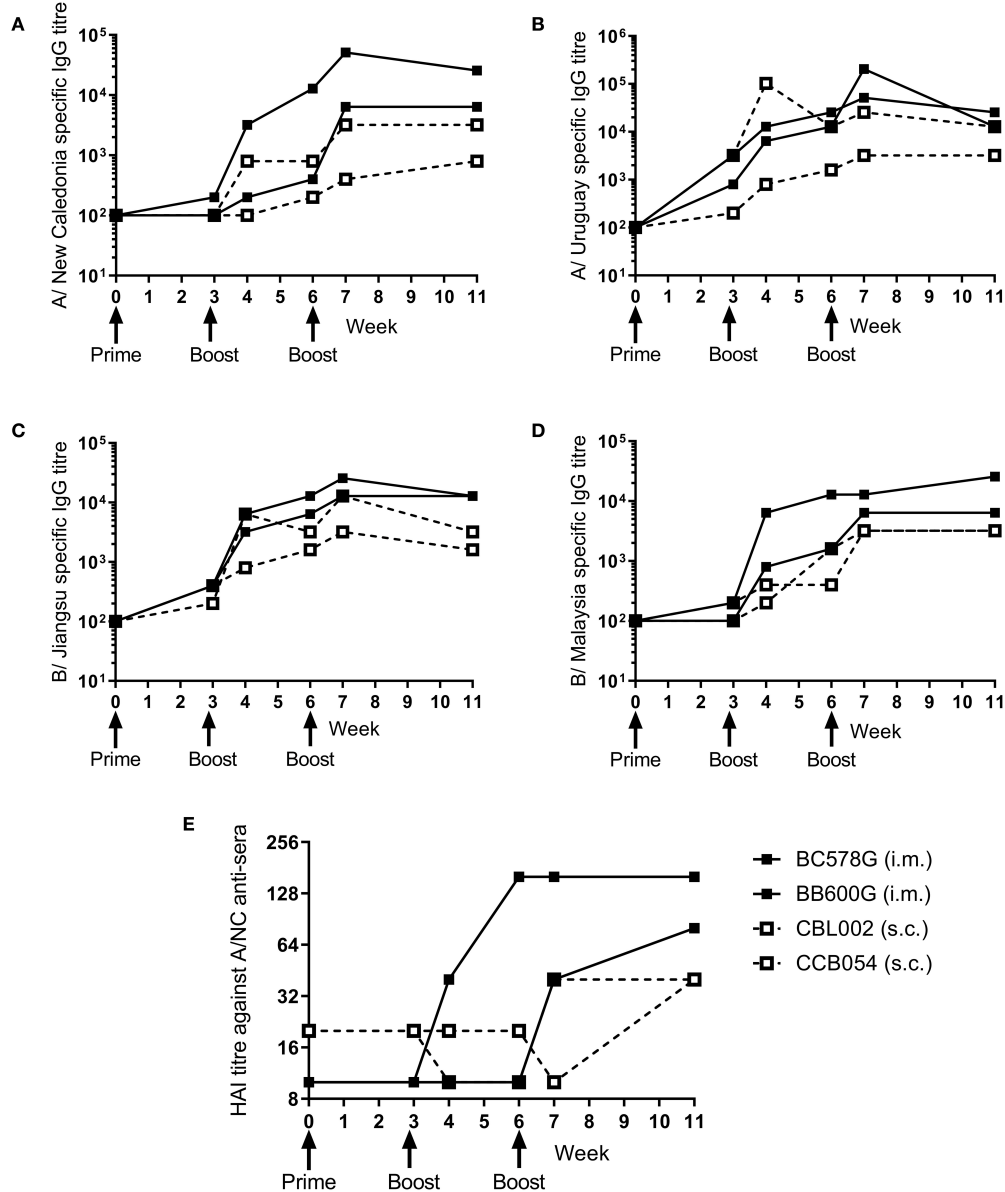
Ciliate produced rHA induces an antibody response in NHP. NHP were immunized three times at 3 weekly intervals with 45 μg each of A/NC **(A)**, A/Uru **(B)**, B/Jia **(C)**, and B/Mal **(D)** by the intramuscular (i.m.) or subcutaneous (s.c.) routes, *n* = 2 animals per route. Blood samples for ELISA **(A–D)** and haemagglutination inhibition **(E)** analysis were collected.

To determine whether the ciliate produced rHA were immunogenic in a range of species, a small number of non-human primates (NHP) were immunized at 3 weekly intervals with a quadrivalent mixture of influenza antigens containing 45 μg each of A/NC, A/Uru, B/Jia and B/Mal by the i.m. or s.c. routes. Two animals were immunized per route. The antibody response to the four antigens was measured by ELISA (Figures 3A–D). There was little response to the prime immunization in any of the animals to any of the antigens. However, at week 6, after the boost immunization, there was detectable antibody responses in all four animals to all 4 antigens. The second boost immunization at 6 weeks elevated this response further. The quality of the antibody response was assessed by HAI assay (Figure 3E), by week 11 an HAI titer against H1N1, but not the other antigens, was detectable in all animals.

### Ciliate Produced rHA Protects Against Infection With H1N1 or B Influenza

**Figure 4:**
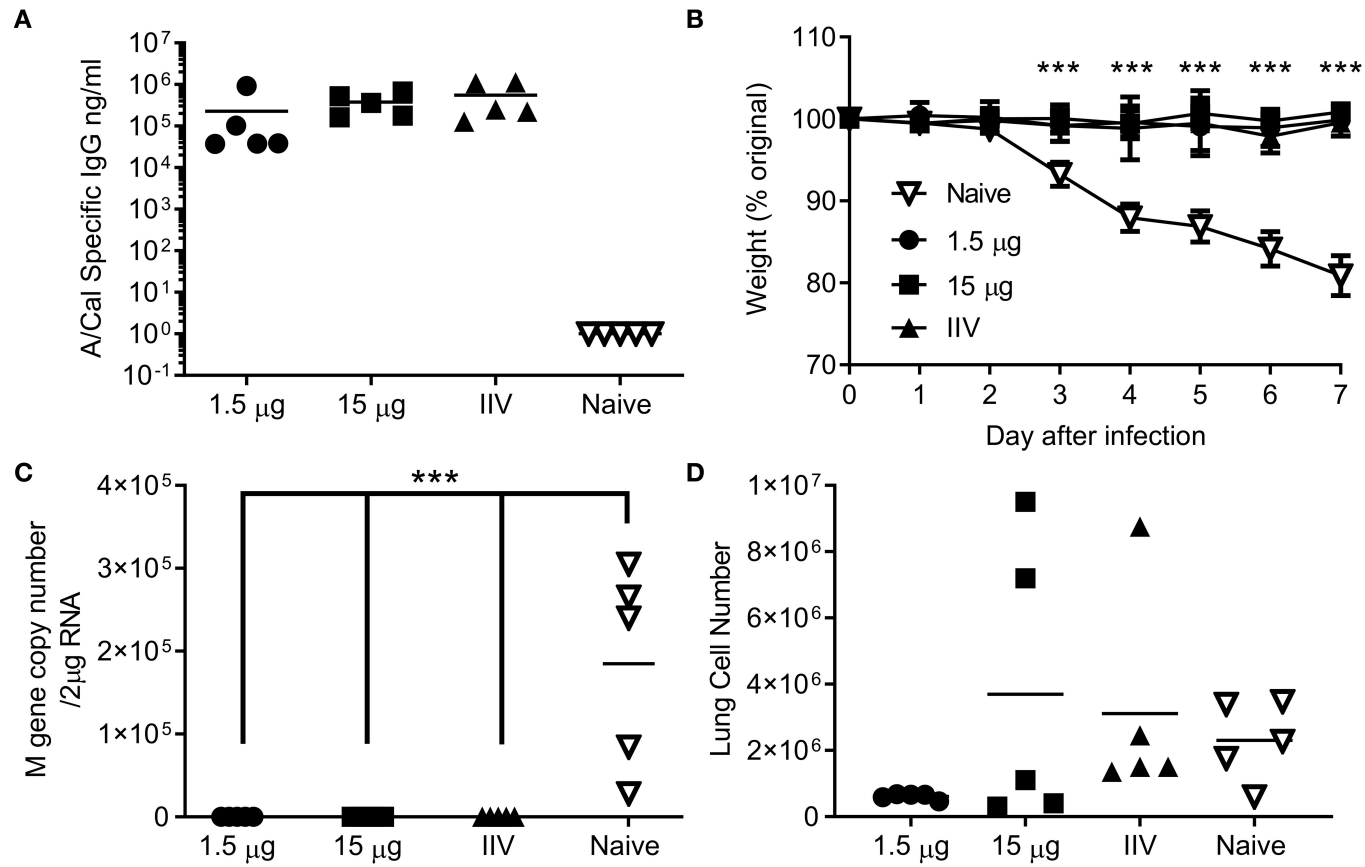
Ciliate produced rHA protects against infection with H1N1 influenza. Mice were immunized three times at 3 weekly intervals with 15 or 1.5 μg ciliate derived A/Cal, or 15 μg control inactivated influenza antigen (IIV) by the intramuscular (i.m.) route. Two weeks after the final immunization, anti-H1N1 antibody titer was measured by ELISA (A). Mice were then challenged with pH1N1 by the intranasal route. Weight change **(B)**, viral load **(C)**, and lung cell number **(D)** were assessed after infection. Points represent individual animals **(A,C,D)** or mean **(B)** of *n* = 5 mice per group, ^***^p < 0.001 by ANOVA and post test.

We wanted to test whether the immune response induced by ciliate produced antigens was protective against infection. Since no significant difference was observed between i.m. and s.c. immunization routes, protection studies were performed upon i.m. immunization, as this is the route in human vaccination. Mice were immunized by the i.m. route in a prime-boost-boost regime with either a high (15 μg) or low (1.5 μg) dose of ciliate produced A/Cal rHA, responses were compared to 15 μg positive control egg derived inactivated influenza antigen (IIV). After 3 doses of vaccine, mice immunized with either the high or low dose of antigen had a high level of HA influenza specific antibody in serum (Figure 4A). Mice were then challenged i.n. with A/Cal pH1N1 strain. Vaccinated mice did not lose weight after infection and there was a significant difference between vaccinated and control mice (Figure 4B). There was also significantly less viral RNA in the lungs of vaccinated mice than control unvaccinated mice (*p* < 0.001, Figure 4C). There were also fewer infiltrating cells (a measure of lung inflammation) in the group vaccinated with 1.5 μg of rHA A/Cal (Figure 4D).

**Figure 5:**
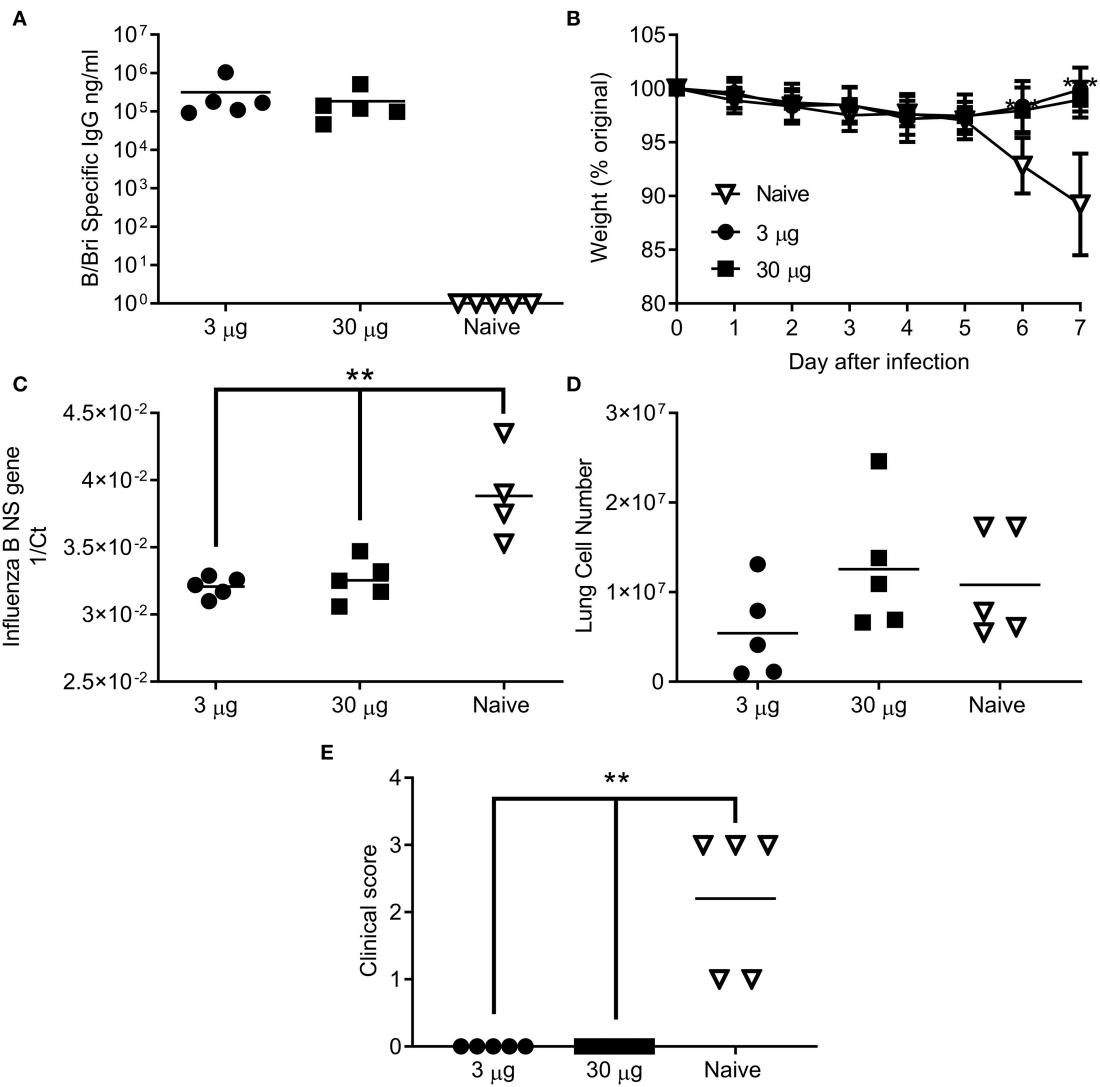
Ciliate produced rHA protects against infection with B/Brisbane influenza. Mice were immunized three times at 3 weekly intervals with 30 or 3 μg ciliate derived B/Bri by the intramuscular (i.m.) route. Two weeks after the final immunization, anti-B/Bri antibody titer was measured by ELISA **(A)**. Mice were then challenged with B/Bri by the intranasal route. Weight change **(B)**, viral load **(C)**, lung cell number **(D)**, and clinical score **(E)** were assessed after infection. Points represent individual animals **(A,C,D,E)** or mean **(B)** of *n* = 5 mice per group, ^**^p < 0.01, ^***^p < 0.001 by ANOVA and post test.

Having seen that the ciliate derived HA antigen was protective, we also tested the protective efficacy of ciliate derived B/Bri against challenge. Mice were immunized in a prime-boost-boost regime with either a high (30 μg) or low (3 μg) dose of ciliate produced B/Bri rHA. After 3 doses of vaccine, mice immunized with either the high or low dose of antigen had a high level of influenza B specific antibody in serum (Figure 5A). Mice were then challenged i.n. with B/Bri influenza strain; there was a significant difference between vaccinated and control mice on days 6 and 7 after infection (*p* < 0.01, Figure 5B). There was significantly less viral RNA in the lungs of vaccinated mice than control unvaccinated mice (*p* < 0.001, Figure 5C). There was no difference in infiltrating cells between the groups (Figure 5D) but the control unvaccinated mice had more signs of disease by clinical score (*p* < 0.01, Figure 5E). Therefore, the ciliate derived antigens were protective against homologous challenge with representative A or B strains of influenza.

### Combination of Antigen With Nod2 Particles Enables Dose Sparing

**Figure 6:**
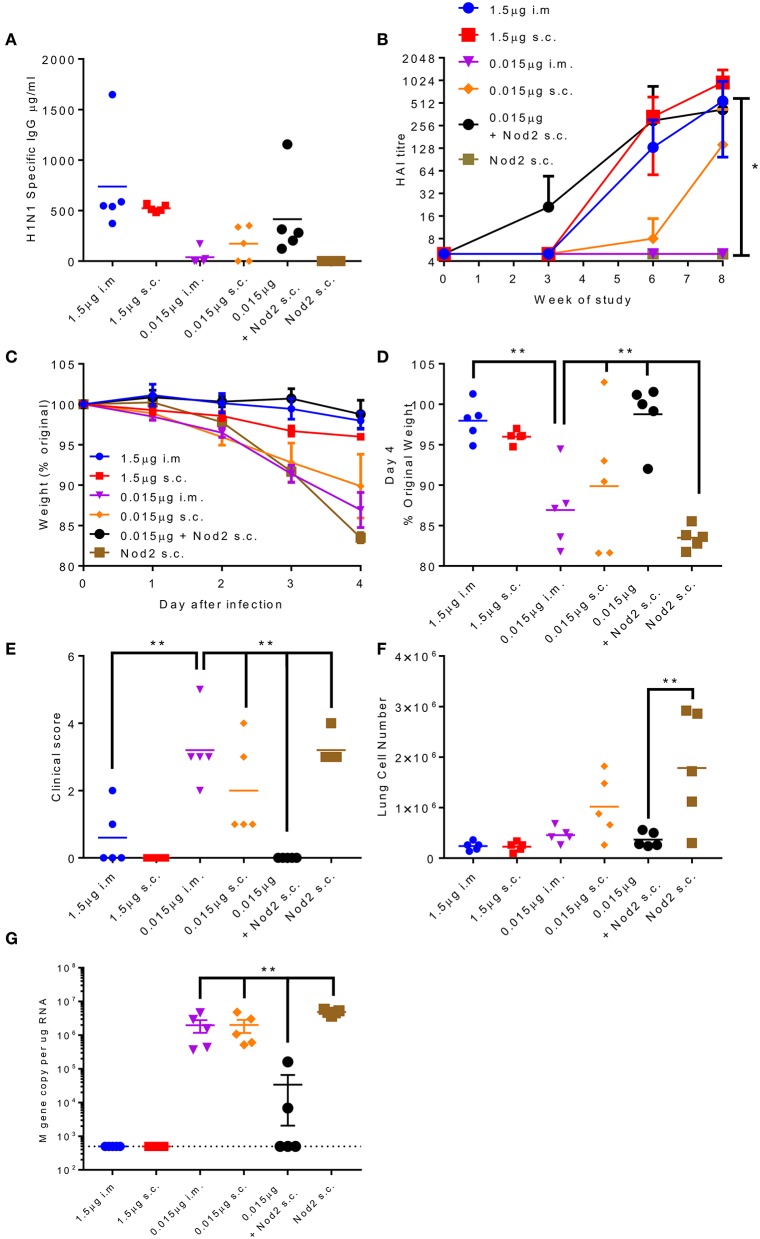
Combination of antigen with PLA-Nod2 particles enables dose sparing. Mice were immunized three times at 3 weekly intervals with 1.5 or 0.015 μg ciliate derived rHA A/Cal by the intramuscular (i.m.) or subcutaneous (s.c.) routes; responses were compared to 0.015 μg antigen loaded onto PLA-Nod2 particles or empty PLA particles. Anti-H1 antibody was measured by ELISA **(A)** or HAI **(B)**. Mice were then challenged with A/Cal by the intranasal route. Weight change over time **(C)** and at d4 **(D)**, clinical score **(E)**, lung cell number **(F)**, and viral load **(G)** were assessed after infection of *n* = 5 mice per group, ^**^p < 0.001 by ANOVA and post test. Points represent individual animals **(A,D,E,F,G)** or mean **(B,C)** of *n* = 5 mice per group, ^*^p < 0.05, ^**^p < 0.01 by ANOVA and post test.

The production of protein antigen is a major constraint in the manufacture of vaccines. Reduction of the amount of protein required per dose could increase coverage and reduce costs. One approach to reduce protein is to incorporate an adjuvant. We have previously shown that the inclusion of Nod2 ligands into poly(lactic acid) particles potentiates their immune properties (32). We wanted to test whether these particles could lead to protection with a smaller dose of antigen. We compared two doses of A/Cal antigen 1.5 and 0.015 μg by the i.m. and s.c. routes; responses were compared between low dose antigen alone and antigen combined with PLA-Nod2 particles for the s.c. route, which has previously been used in nanoparticle studies (23). Mice were immunized three times in a prime-boost-boost regime. At week 8 the low dose antigen induced some anti-influenza antibody, but less than the high groups (Figure 6A). Inclusion of the PLA-Nod2 particles increased the response to the low dose antigen. The Nod2 plus antigen and the high dose antigen groups also induced an HAI response more rapidly than the low dose groups, with a significantly greater HAI titer in the Nod plus antigen group at week 8 (*p* < 0.05, Figure 6B). To test whether there was an effect on protective efficacy, mice were challenged with A/Cal pH1N1 strain i.n. after the final boost dose. The inclusion of PLA-Nod2 particles significantly reduced weight loss at day 4 after infection compared to the low dose antigen alone groups (Figures 6C,D). The high dose of antigen (1.5 μg) was significantly more protective than the low dose (0.015 μg). The antigen plus adjuvant immunized group also had fewer signs of disease (Figure 6E), and lower cell infiltration into the lungs (Figure 6F), than low dose antigen alone. There was also significantly lower viral load in the group immunized with antigen plus adjuvant compared to the low dose antigen alone groups (Figure 6G). Mice inoculated with PLA-Nod2 particles without vaccine antigen, the negative control group, lost weight at day 4 after infection compared to the low dose antigen alone groups (Figures 6C,D).

## Discussion

In the current study we show that influenza HA can be produced in the ciliate *T. thermophila*. The ciliate derived antigen was immunogenic and able to protect against infection with influenza A or B viruses in mice. In a small study with limited animal numbers, we also observed immunogenicity of the material in macaques. One consideration for the future is to evaluate immunogenicity in ferret models, which would enable the evaluation of protection against transmission. This would then be a precursor for generating GMP grade material for a clinical trial.

We also evaluated the dose sparing effect by combining the *Tetrahymena* derived antigen with PLA-Nod2 particles. Nanoparticle and microparticle delivery systems have received attention for a range of biological applications and including showing promise as vaccine vehicles. A variety of polymers exists from which particles for drug delivery can be prepared, of which poly(lactic-co-glycolic acid) (PLGA) and poly(lactic acid) (PLA) are the most commonly studied (33). They have been licensed for human use for both physical functions, as sutures, bone implants and screws and as implants for sustained drug delivery, taking advantage of their biodegradable and biocompatible properties. PLA and PLGA have been extensively studied for vaccine formulation with a body of literature demonstrating their advantages for antigen delivery. PLA and PLGA particles can be adapted to degrade over a large kinetic range and are able to act as depots from which antigen or bioactive molecules are gradually released. The synthesis of PLA i-Particles^®^ is based on the nanoprecipitation of poly (lactic acid polymer) in aqueous phase, a surfactant free process, which allows the reproducible production of safe submicron particles, with homogenous diameter (34). This process permits the encapsulation of any kind of hydrophobic molecules, such as PRR (Pattern Recognition Receptors) ligands without impairing their colloidal properties (size, surface charges). Being negatively charged, any protein moieties could be passively adsorbed, thus those particles can combine antigen and immunostimulatory molecule delivery, leading to versatile biodegradable multifunctional particulate vaccine vehicle, allowing the delivery of vaccines through systemic, and mucosal routes (35). Indeed, the preferential uptake of particles by Dendritic Cells (DCs) can induce a strong up regulation of DC maturation and the enhancement of pro-inflammatory cytokine, thus improving immunogenicity. For instance, encapsulation of NOD2 ligand has been able to favor mucosal immune responses after a sub cutaneous administration of PLA particles (36) and this ligand was used in the current study. One advantage of including adjuvant is dose reduction, combining rHA antigen with PLA-Nod2 particles led to a similar levels of antibody protection with much lower levels of antigen, this may be advantageous in a pandemic.

Here we show that *Tetrahymena* can be used to produce immunogenic influenza antigens. One advantage of recombinant protein-based influenza vaccines is the avoidance of infectious viral propagation which normally requires increased biocontainment, allowing the use of lowest biosafety level laboratories and facilities for the cultivation of *Tetrahymena*. Since *Tetrahymena* can be cultivated in standard bioreactor vessels (16), the *Tetrahymena* based expression system enabling large scale manufacturing in common, market-standard contract manufacturing facilities. In consequence, production of subunit vaccines can be rapidly scaled up without concerns about the supply of embryonated eggs. This manufacturing flexibility could be advantageous compared to other cell culture approaches, for example cell culture-based technologies using adherent cell lines, such as MDCK or Vero cells, are inherently difficult to scale up because of the requirements for an attachment surface (37). Likewise, the slow growth of insect cells (12–24 h generation time) and the sensitivity of insect cells to the stress linked to the mechanical agitation in stirred tank reactors (38) might present a challenge for large scale manufacturing. A further limitation of insect cells for the production of recombinant influenza vaccines is the large volume of virus needed on scale-up and the time sensitivity of harvest to avoid potential cell lysis and degradation of expressed proteins (39). More work is still needed to demonstrate that the advantages of using *Tetrahymena* would be realized in a large-scale manufacturing setting. Proteins expressed in this system should be biologically safe as there is no evidence that *T. thermophila* harbors any viruses pathogenic to humans (16), though detailed GMP work up, including a full characterization of any possible adventitious agents and the stability of the platform, would be required before human vaccine manufacture could be performed.

In the current pre-clinical study, we demonstrate immunogenicity and protective efficacy of *Tetrahymena* rHA. We have not looked at comparative efficacy compared to other platforms for generating influenza vaccines, and the animal models may not necessarily enable us to explore the advantages, which are associated with manufacturing cost, speed and scale-up. But based on the data presented, this study supports the further development and testing of the *Tetrahymena* platform for vaccine antigens.

## Data Availability Statement

The datasets generated for this study are available on request to the corresponding author.

## Ethics Statement

Mouse study UK: Studies were approved by Imperial College London, AWERB. Mouse study Italy: Studies were approved by the Italian Ministry of Health with authorization n° 1004/2015-PR, 22 Sept 2015. NHP study was approved and accredited under statement number (A15 007) by the ethics committee Comité d'Ethique en Expérimentation Animale du CEA, registration number 44 for the French Ministry of Research.

## Author Contributions

KJ: conceptualization, investigation, resources, and writing—original draft. MH: conceptualization, funding acquisition, and writing—original draft. CS, EK, DB, FF, VC, and CT: investigation. AC: funding acquisition, investigation, and writing—review and editing. DM: supervision and funding acquisition. EM and RL: supervision. CC and CP: resources. BV: conceptualization, funding acquisition, resources, and writing—review and editing. JT: conceptualization, supervision, funding acquisition, and writing—original draft.

### Conflict of Interest

KJ and MH work for Cilian which developed the ciliate platform and has a commercial interest. BV and CP work for Adjuvatis which owns the adjuvant platform and have a commercial interest. Both of these parties supplied material and were not involved in data analysis or interpretation. EM works for Vismederi srl, which analyses responses influenza vaccines and infection, Vismederi analyzed data from the project. The remaining authors declare that the research was conducted in the absence of any commercial or financial relationships that could be construed as a potential conflict of interest.

## Supplementary Material

Figure S1 SDS-PAGE of ciliate produced, purified rHA from influenza virus A and B strains. Each purified rHA was analyzed by SDS-PAGE and silver staining **(A,C,E,G)** and Western blot **(B,D,F,H)**. A/NC: ciliate derived rHA A/NewCaledonia/20/99; A/Uru: ciliate derived rHA A/Uruguay/716/2007; B/Jia: ciliate derived rHA B/Jiangsu/10/2003; B/Mal: ciliate derived rHA B/Malaysia/2506/2004; PC: positive control (A/NewCaledonia/20/99 antigen (01/614, NIBSC); A/Brisbane/10/2007 (08/278, NIBSC); B/Jiangsu/10/2003 (04/202, NIBSC); B/Malaysia/2506/2004 (08/184, NIBSC); M, molecular weight marker).

Table S1 Growth and purification of different rHA antigens.
