# Supplementary figures and images for: Recombinant Haemagglutinin Derived From the Ciliated Protozoan Tetrahymena thermophila Is Protective Against Influenza Infection
Source: Front Immunol. 2019 Nov 13;10:2661. doi: 10.3389/fimmu.2019.02661 (PMC6863932; doi:10.3389/fimmu.2019.02661)

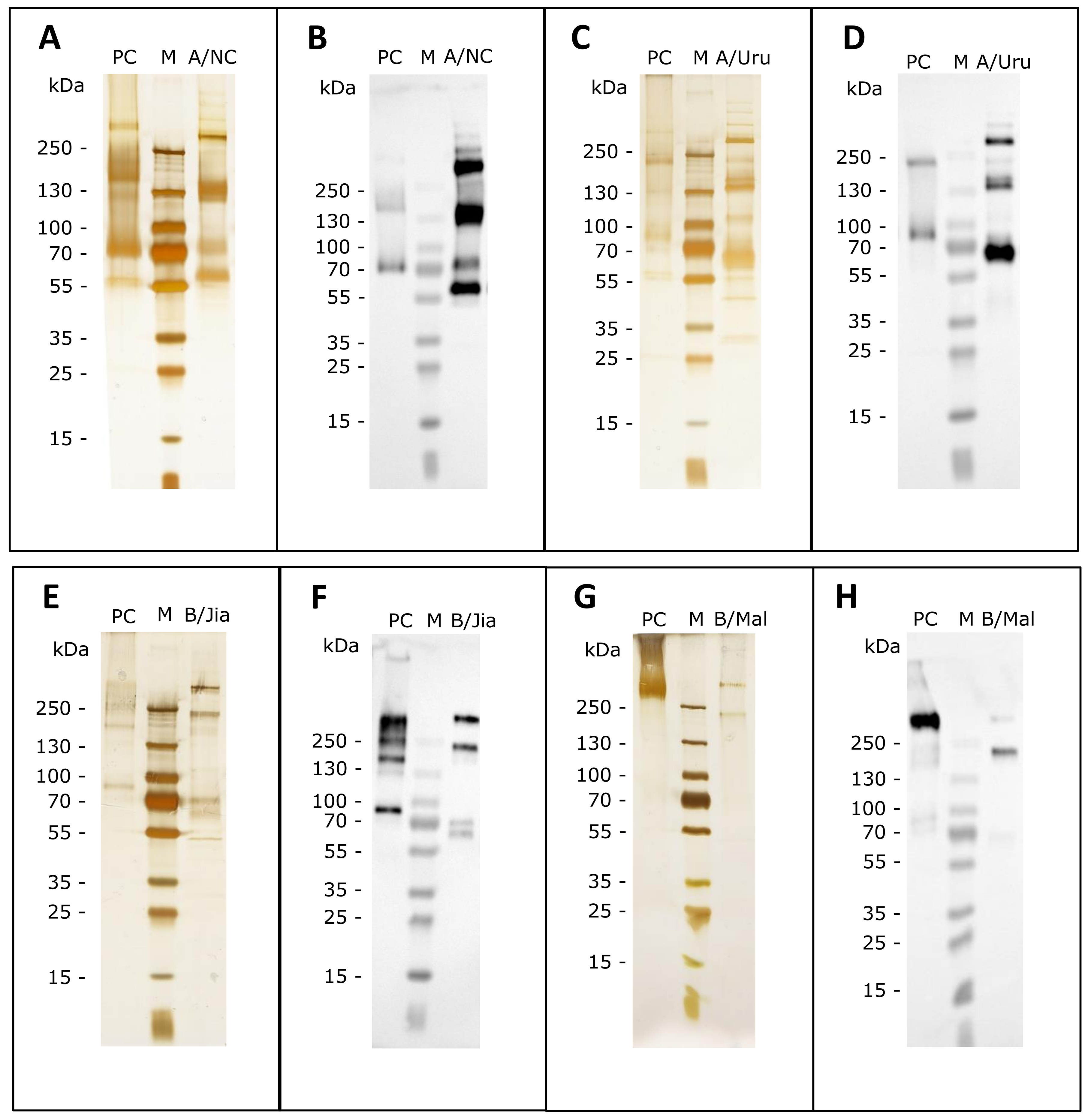

Supplement: Figure S1 — SDS-PAGE of ciliate produced, purified rHA from influenza virus A and B strains. Each purified rHA was analyzed by SDS-PAGE and silver staining (A,C,E,G) and Western blot (B,D,F,H). A/NC: ciliate derived rHA A/NewCaledonia/20/99; A/Uru: ciliate derived rHA A/Uruguay/716/2007; B/Jia: ciliate derived rHA B/Jiangsu/10/2003; B/Mal: ciliate derived rHA B/Malaysia/2506/2004; PC: positive control (A/NewCaledonia/20/99 antigen (01/614, NIBSC); A/Brisbane/10/2007 (08/278, NIBSC); B/Jiangsu/10/2003 (04/202, NIBSC); B/Malaysia/2506/2004 (08/184, NIBSC); M, molecular weight marker). [file Image_1.TIF]
