# Supplementary material for: Recombinant Haemagglutinin Derived From the Ciliated Protozoan Tetrahymena thermophila Is Protective Against Influenza Infection
Source: Front Immunol. 2019 Nov 13;10:2661. doi: 10.3389/fimmu.2019.02661 (PMC6863932; doi:10.3389/fimmu.2019.02661)
Supplement: Table S1 — Growth and purification of different rHA antigens. [file Table_1.DOCX]

**Table S1: Growth and purification of different rHA antigens**

| **Antigen** | **A/Cal** | **A/NC** | **A/Uru** | **B/Bri** | **B/Jia** | **B/Mal** |
| --- | --- | --- | --- | --- | --- | --- |
| **Growth Medium** | SPO medium supplemented with 1 % Casein hydrolysate | SPP medium supplemented with 1 % Casein hydrolysate | SPP medium supplemented with 1 % Casein hydrolysate | SPO medium supplemented with 1 % Casein hydrolysate | SPP medium supplemented with 1 % Casein hydrolysate | SPP medium supplemented with 1 % Casein hydrolysate |
| **Fermentation scale** | 400 mL | 50 L | 50 L | 400 mL | 400 mL | 400 mL |
| **Induction CdCl_2_** | 94 µM | 47 µM | 47 µM | 94 µM | 94 µM | 94 µM |
| **Solubilisation** |  |  |  |  |  |  |
| **Buffer A**  **(Solubilisation buffer)** | 20 mM ethanolamine, 5 % Glycerol, 2.5mg/L E-64, 25 mM NaCl, 0.5 % Triton X-100, pH 11.3 at 4°C | 10 mM ethanolamine, 5 % Glycerol, 2.5 mg/L E-64, 10 mM NaCl, 0.1 % Tergitol NP-9, pH 10.5 at 4°C | 15 mM ethanolamine,  5 % Glycerol, 2.5 mg/L E-64,  10 mM NaCl, 0.1 % Tergitol NP-9, pH 10.5 at 4°C | 20 mM ethanolamine, 5 % Glycerol, 2.5 mg/L E-64, 25 mM NaCl, 0.76 % Triton X-100, pH 11 at 4°C | 15 mM ethanolamine,  5 % Glycerol, 2.5 mg/L E-64,  0.1 % Tergitol NP-9, pH 10.5 at 4°C | 10 mM ethanolamine,  5 % Glycerol, 2.5 mg/L E-64,  10 mM NaCl,  0.1 % Tergitol NP-9, pH 10.5 at 4°C |
| **Buffer B**  **(Buffer adjustment)** | 20 % sodium dihydrogen phosphate | 15% Tris,  for final conc. of 20 mM Tris | 5 mM sodium phosphate | 20 % sodium dihydrogen phosphate | 5 mM sodium phosphate | 15% Tris,  for final conc. of 20 mM Tris |
| **1^st^ columns** | Capto SP ImpRes, ø 1.6 cm x 12.5 cm, 25 mL | Capto Q, ø 1.6 cm x 12.5 cm, 25 mL | CHT Hydroxyapatite ø 1.6 cm x 12.5 cm, 25 mL | Capto SP ImpRes, ø 1.6 cm x 12.5 cm, 25 mL | CHT Hydroxyapatite ø 1.6 cm x 12.5 cm, 25 mL | Capto Q, ø 1.6 cm x 12.5 cm, 25 mL |
| **Buffer C**  **(Binding + wash buffer)** | 20 mM sodium phosphate,  5 % Glycerol, 0,15 % Triton X-100, pH 7.13 | 20 mM Tris,  5 % Glycerol, 1 M NaCl, 0.05 % Tergitol NP-9, pH 8.6 | 15 mM ethanolamine,  5 % Glycerol,  10 mM NaCl,  5 mM sodium phosphate,  0.1 % Tergitol NP-9, pH 10 at 4°C | 20 mM sodium phosphate, 5 % Glycerol, 0,15 % Triton X-100, pH 7.15 | 15 mM ethanolamine,  5 % Glycerol,  10 mM NaCl,  5 mM sodium phosphate,  0.1 % Tergitol NP-9, pH 10 at 4°C | 20 mM TRIS,  5 % Glycerol,  10 mM NaCl,  0.1 % Tergitol NP-9, pH 8.6 |
| **Buffer D**  **(Elution buffer)** | 20 mM sodium phosphate,  5 % Glycerol, 0,15 % Triton X-100, 1 M NaCl, pH 7.13 | 20 mM Tris,  5 % Glycerol, 1 M NaCl, 0.05 % Tergitol NP-9, pH 8.6 | 15 mM ethanolamine,  5 % Glycerol,  10 mM NaCl, 100 mM sodium phosphate,  0.1 % Tergitol NP-9, pH 10 | 20 mM sodium phosphate, 5 % Glycerol, 0,15 % Triton X-100, 1 M NaCl, pH 7.15 | 15 mM ethanolamine,  5 % Glycerol,  10 mM NaCl, 100 mM sodium phosphate,  0.1 % Tergitol NP-9, pH 10 | 20 mM TRIS,  5 % Glycerol,  1 M NaCl,  0.1 % Tergitol NP-9, pH 8.6 |
| **Buffer E**  **(Buffer adjustment)** | PBS with 25 mM NaCl, pH 7.4 | ½fold PBS, pH 7.4 at 4°C | 10 mM sodium phosphate,  5 % Glycerol,  10 mM NaCl, 0.05 % Tergitol NP-9, pH 7 | PBS with 25 mM NaCl, pH 7.4 | 20 mM Tris,  5 % Glycerol,  10 mM NaCl, 0.05 % Tergitol NP-9, pH 8,6 | ½fold PBS, pH 7.4 at 4°C |
| **2^nd^ columns** | Fetuin-Agarose, ø 1.6 cm x 5 cm, 10 mL | Fetuin-Agarose, ø 1.6 cm x 3.35 cm, 6.7 mL | Capto Q, ø 1.6 cm x 12.5 cm, 25 mL | Fetuin-Agarose, ø 1.6 cm x 5 cm, 10 mL | Capto Q, ø 1.6 cm x 12.5 cm, 25 mL | Fetuin-Agarose, ø 1.6 cm x 6 cm, 12 mL |
| **Buffer F**  **(Binding + wash buffer)** | ½fold PBS, pH 7.4 at 4°C | ½fold PBS, pH 7.4 at 4°C | 10 mM sodium phosphate,  5 % Glycerol,  10 mM NaCl, 0.05 % Tergitol NP-9, pH 7 | ½fold PBS, pH 7.4 at 4°C | 20 mM TRIS,  5 % Glycerol,  10 mM NaCl,  0.05 % Tergitol NP-9, pH 8.6 | ½fold PBS, pH 7.4 at 4°C |
| **Buffer G**  **(Elution buffer)** | PBS with 2M NaCl, 0.15 % Triton X-100, pH 7.4 | PBS with 2 M NaCl,  0.1 % Tergitol NP-9, pH 7.4 | 10 mM sodium phosphate,  5 % Glycerol,  1 M NaCl,  0.05 % Tergitol NP-9, pH 7 | PBS with 2M NaCl, 0.15 % Triton X-100, pH 7.4 | 20 mM TRIS,  5 % Glycerol,  1 M NaCl,  0.05 % Tergitol NP-9, pH 8.6 | PBS with 2 M NaCl,  0.1 % Tergitol NP-9, pH 7.4 |
| **Buffer H**  **(Buffer adjustment)** | 20 mM sodium phosphate, pH 7.15 | N/A | N/A | 20 mM sodium phosphate, pH 7.25 | ½fold PBS, pH 7.4 at 4°C | N/A |
| **3^rd^ columns** | Capto SP ImpRes HiScreen, ø 0.77 cm x 10 cm, 4.7 mL | N/A | Capto SP ImpRes, ø 1.6 cm x 12.5 cm, 25 mL | Capto SP ImpRes HiScreen, ø 0.77 cm x 10 cm, 4.7 mL | Fetuin-Agarose, ø 1.6 cm x 3.5 cm, 7 mL | N/A |
| **Buffer I**  **(Binding + wash buffer)** | 20 mM sodium phosphate, 0.05 % Tween 20, pH 7.15 | N/A | 10 mM sodium phosphate,  5 % Glycerol,  10 mM NaCl, 0.05 % Tergitol NP-9, pH 7 | 20 mM sodium phosphate, 0.05 % Tween 20, pH 7.25 | ½fold PBS, pH 7.4 at 4°C | N/A |
| **Buffer J**  **(Elution buffer)** | 20 mM sodium phosphate, 0.05 % Tween 20, 1 M NaCl, pH 7.15 | N/A | 10 mM sodium phosphate,  5 % Glycerol,  1 M NaCl,  0.05 % Tergitol NP-9, pH 7 | 20 mM sodium phosphate, 0.05 % Tween 20, 1 M NaCl, pH 7.25 | PBS with 2 M NaCl,  0.75 % Tergitol NP-9, pH 7.4 | N/A |
| **Buffer K (final buffer)** | PBS with 25 mM NaCl, 0.05 % Tween 20, pH 7.4 | PBS, 0.05 % Tergitol NP-9, pH 7.4 | PBS, 0.05 % Tergitol NP-9, pH 7.4 | PBS with 25 mM NaCl,  0.05 % Tween 20, pH 7.4 | PBS, 0.05 % Tergitol NP-9, pH 7.4 | PBS, 0.05 % Tergitol NP-9, pH 7.4 |
